# Photoluminescence of CdSe/ZnS quantum dots in nematic liquid crystals in electric fields

**DOI:** 10.3762/bjnano.9.145

**Published:** 2018-05-23

**Authors:** Margarita A Kurochkina, Elena A Konshina, Daria Khmelevskaia

**Affiliations:** 1 Center of Information and Optical Technologies, ITMO University, Kronverksky pr. 49, Saint-Petersburg, 197101, Russia; 2 Department of Optical Physics and Modern Natural Science, ITMO University, Kronverksky pr. 49, Saint-Petersburg, 197101, Russia

**Keywords:** aggregation, decay time, liquid crystal, luminescence intensity, orientation

## Abstract

We have experimentally investigated the effect of the reorientation of a nematic liquid crystal (LC) in an electric field on the photoluminescence (PL) of CdSe/ZnS semiconductor quantum dots (QDs). To the LC with positive dielectric anisotropy, 1 wt % QDs with a core diameter of 5 nm was added. We compared the change of PL intensity and decay times of QDs in LC cells with initially planar or vertically orientated molecules, i.e., in active or passive LC matrices. The PL intensity of the QDs increases four-fold in the active LC matrix and only 1.6-fold in the passive LC matrix without reorientation of the LC molecules. With increasing electric field strength, the quenching of QDs luminescence occurred in the active LC matrix, while the PL intensity did not change in the passive LC matrix. The change in the decay time with increasing electric field strength was similar to the behavior of the PL intensity. The observed buildup in the QDs luminescence can be associated with the transfer of energy from LC molecules to QDs. In a confocal microscope, we observed the increase of particle size and the redistribution of particles in the active LC matrix with the change of the electric field strength. At the same time, no significant changes occurred in the passive LC matrix. With the reorientation of LC molecules from the planar in vertical position in the LC active matrix, quenching of QD luminescence and an increase of the ion current took place simultaneously. The obtained results are interesting for controlling the PL intensity of semiconductor QDs in liquid crystals by the application of electric fields.

## Introduction

Colloidal quantum dots (QDs) are a special kind of nanocrystals. These nanoparticles (NPs) of spherical shape are unique luminophores due to the dimensional dependence of the optical properties. The small dimensions of QDs (of the order of 1–10 nm) make it possible to integrate QDs relatively easily into hybrid structures and composite materials. Quantum dots demonstrate unique properties such as high quantum yield, narrow symmetric luminescence peak and high photostability, which are used in optical molecular sensor systems [1], bioanalysis [2], solar cells [3–4], and light-emitting devices [5]. The application of external electric fields to semiconductor NPs significantly changes their optical properties [6–10]. To control the properties of NPs, they are introduced into a passive or active matrix interacting with them.

Liquid crystals can be an active matrix for NPs. The dielectric and optical properties of a liquid crystal (LC) vary under electromagnetic fields. The control of the QD luminescence in an LC matrix has both scientific and practical interest. The photoluminescence (PL) of liquid crystals doped with silver NPs, carbon nanotubes and quantum semiconductor nanoparticles were discussed in [11–12]. The control of PL of a nematic liquid crystal doped with QDs by applying external electric fields was demonstrated in [13–14]. In order to realize various applications of quantum dots in the LC matrix, it is necessary to control and stabilize dispersions of nanoparticles in different phases of the liquid crystal [15–17].

We have investigated spectral luminescence properties of the nematic LCs with a positive dielectric anisotropy doped with semiconductor CdSe/ZnS quantum dots [18–20]. The luminescence quenching of a planar oriented liquid crystal depended not only on the size but also on the concentration of QDs [18]. The PL intensity of the LC suspension doped with CdSe/ZnS QDs having a core size of 3.5 nm in the planar oriented cell decreased exponentially with an increase of the electric field strength [20]. We showed that the decrease of PL intensity correlated with the increase of dielectric losses in a nematic associated with ionic impurities. Luminescence quenching of a nematic doped with CdSe/ZnS QDs is due to nonradiative transfer of energy from LC molecules to QDs [20].

The main goal of this work was to investigate the dependence of luminescence properties of CdSe/ZnS quantum dots in a LC matrix on the reorientation of the director field under the action of an electric field. We have used a nematic liquid crystal with positive dielectric anisotropy as the LC matrix. The liquid crystal doped with QDs was aligned in parallel (a vertical orientation) or perpendicular (a planar orientation) to the electric field vector in the initial state of the LC cell. When an electric field is applied, the reorientation of molecules occurs only in the LC cell with initially planar orientation. This LC cell acts as an active LC matrix. The LC cell with a vertical orientation is a passive LC matrix, since LC molecules do not change their orientation. We have studied intensity and a decay time of QD photoluminescence as well as the change of particle size and distribution of QDs and ion current in the LC matrix.

## Experimental

We used hydrophobic CdSe/ZnS quantum dots with a core diameter of 5 nm obtained from Belarusian State University (Minsk). The shell of ZnS with wider band gap prevents a degradation of the CdSe core by passivation of broken bonds on its surface. Quantum dots were covered with а layer of surface-active trioctylphosphine oxide (TOPO) molecules. The nematic alkylcyanobiphenyl-based liquid crystal ZhK-1289 (NIOPIK, Moscow, Russia) with dielectric constants 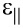 = 16.0 and 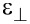 = 5.5, and the isotropic–nematic transition temperature of 62 °C has been used in the study. The dry QDs were mixed with LC for 30 min in an ultrasonic bath. The concentration of QDs was 1 wt %.

We have used plane-parallel LC cells consisting of two quartz substrates with a fixed gap of about 33 μm. Both substrates were coated by cathode sputtering with transparent conductive electrodes based on indium tin oxide. Orienting layers of rubbed polyimide and chromolane were used to obtain a planar or vertical orientation of the LC molecules, respectively. The orientation of the LC after filling the cells was checked using an optical polarization microscope. To obtain the absorption spectra we have used a UV-3600 spectrophotometer (Shimadzu, Japan). To study the QD luminescence a confocal laser scanning microscope LSM 710 (Cаrl Zeiss, Germany) with an excitation light wavelength 405 nm was used. We have used a MicroTime 100 laser scanning microscope (PicoQuant, Germany) to study the kinetics of the luminescence decay of QDs. The semiconductor laser with a frequency of 10 MHz and pulse duration of ca. 80 ps at a wavelength of 409 nm was used in the experiments. Size and distribution of the particles in the LC matrix were studied using the ToupView software. The average area of QDs agglomerates (mathematical expectation) is calculated as

[1]
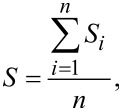


where *S_i_* is the area of the *i*-th particle and *n* is the total number of agglomerates. To measure the conductivity of the LC doped with QDs, we have used a E4980A LCR meter (Keysight Technologies, USA).

## Results and Discussion

**Figure 1:**
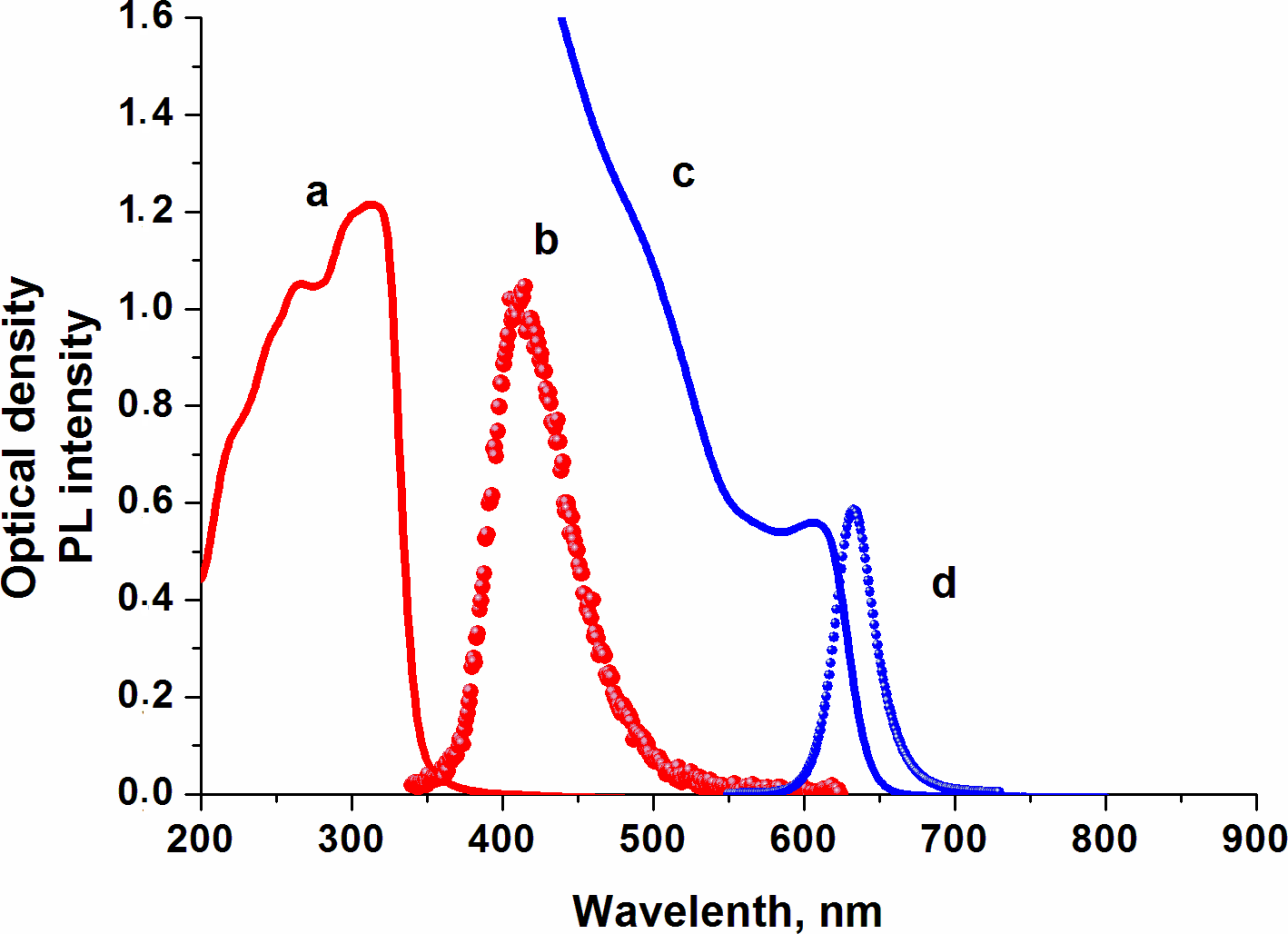
Absorption (solid lines) and luminescence (dotted lines) spectra of (a,b) a nematic liquid crystal and (c,d) CdSe/ZnS quantum dots core diameter of 5 nm. The excitation wavelength is 320 nm.

In Figure 1 the absorption and luminescence spectra of nematic LC and CdSe/ZnS QDs are shown. The excitation wavelength was 320 nm.

The ZhK-1289 nematic is a mixture of alkylcyanobiphenyl molecules with different alkyl terminal groups. The absorption spectrum of the liquid crystals has two pronounced peaks at wavelengths of 265 nm and 314 nm. These are the bands of the monomers and dimers. Most probably, the band with a maximum of about 414 nm is associated to the excimer luminescence of the LC monomers and dimers [21]. The peak of the maximum luminescence intensity of QDs with 5 nm core diameter is at the wavelength of 630 nm.

**Figure 2:**
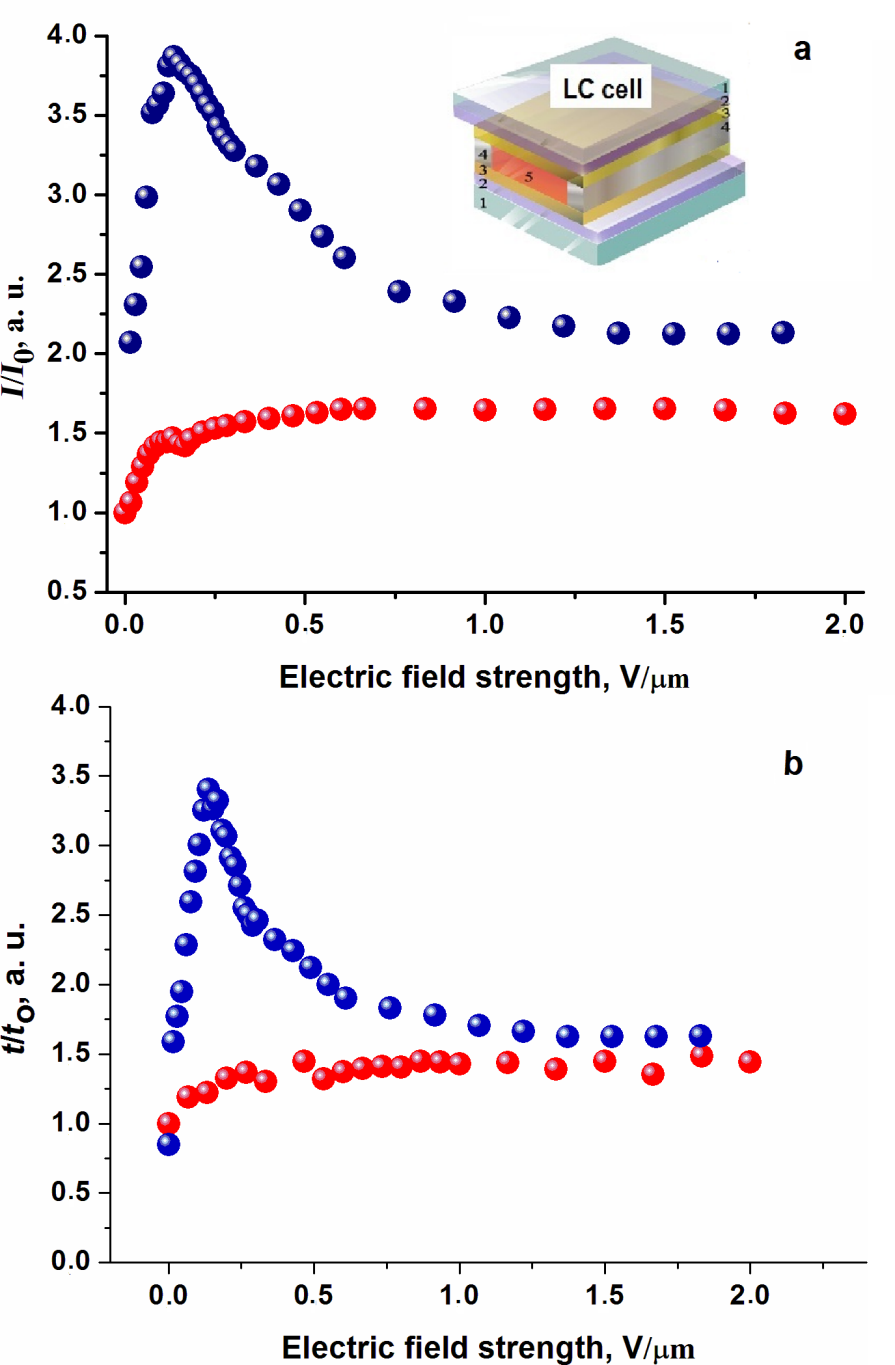
Relative intensities *I*/*I*_0_ (a) and decay times *t*/*t*_0_ (b) of the QD photoluminescence as functions of the electric field strength applied to LC cells with a planar (blue circles) and vertical (red circles) alignment. The insert shows the structure of a LC cell: 1 - quartz substrates, 2 - ITO electrode, 3 - orienting layer, 4 - spacers, 5 - liquid crystal layer.

The quantum yield of the semiconductor NPs luminescence strongly depends on the polarity of the surrounding molecules, the electrostatic properties, the polarizability and the dipole moment of the nanoparticles. The enhancement of the QD luminescence results from the transfer of energy, when the channels of nonradiative deactivation of QDs are eliminated [22]. The polarity of the surrounding molecules affects the optical properties of the QDs. The interaction between CdSe/ZnS and organic molecules can lead to extinction or enhancement of the QD luminescence. Figure 2a shows the relative intensity (*I*/*I*_0_) of the QD luminescence as a function of the electric field strength. The relative PL intensity of the QDs increased by four times when an electric field strength of 0.25 V/μm was applied to the LC cell with an initially planar orientation. With the increase of electric field strength quenching of QD luminescence occurred. The PL intensity decreases to the minimal level at a field strength of about 1.5 V/μm. The increase in the PL intensity of QDs at low electric field strength can arise from the generation of excitons in LC molecules surrounding the QDs and the subsequent Förster resonance energy transfer from the LC molecules (donor) to QDs (acceptor). The NPs spectra can shift and the overlap of the wave functions of electrons and holes can decrease because of a change of the boundary conditions. The luminescence quenching of QDs in the active LC matrix with an increase in the electric field strength occurs when the position of the LC director with respect to the electric field vector changes. The parallel component of the dielectric constant of the LC also changes. The intensity of QDs luminescence increased only 1.6-times in the passive LC matrix (Figure 2a). In this case, the director of LC is parallel to the electric field vector in the LC cell and does not change its orientation. Therefore, the dielectric properties of the LC do not change with increasing electric field strength. As a result, the luminescence intensity remained at the previously achieved level. We assume that the processes of buildup and quenching of the QDs luminescence can occur simultaneously in a passive LC matrix. In the active LC matrix they occur successively and depend on the electric field strength. This confirms the existence of a peak in the curve of PL intensity (Figure 2a).

Figure 2b shows the relative decay times (*t*/*t*_0_) of QD luminescence in the active and passive LC matrices as a function of the electric field strength. Time-resolved luminescence spectroscopy is a quantitative tool for the analysis of the dynamics of photoexcitation in colloidal semiconductor quantum dots [23]. The decay time in the active LC matrix increases by a factor of 3.4 and then begins to decrease with the reorientation of LC molecules to a vertical position due to the increase of field strength. The relative decay time of QD luminescence in the passive LC matrix increased only by 40%, and then did not change with an increase in the electric field strength. The luminescence decay of QDs in the active LC matrix is ca. 54% in Figure 2a. At the same time, the relative decay time decreases by only about 47% (Figure 2b). This means that in 54% of the QDs luminescence has been quenched completely, while in ca. 47% the luminescence has been conserved. The changes in the relative decay time in Figure 2b are alike to those of the PL in Figure 2a. At increasing electric field strength, the PL intensity of the QDs in the active matrix falls to the QDs luminescence level in the passive LC matrix in Figure 2a. The change in the decay time of the luminescence of the QD in the LC in Figure 2b has the same tendency.

The luminescence decay of the quantum dots exhibits a bi-exponential dependence on two independent, but spectrally overlapping, processes. The first process involves the recombination of the so-called “internal” exciton with energy close to the width of the band gap and a decay time of about 20–30 ns, depending on the size of the quantum dots. The second process is associated with recombination of the so-called “charged” exciton, which appears in a charged nanoparticle for a period of time until one of the photoexcited electrons is trapped. The average decay time of a “charged” exciton is about 1–3 ns [24–25]. The increase in the relative decay time of the QDs luminescence can be related to one of the components of the bi-exponential approximation, or to the redistribution of their contributions.

**Figure 3:**
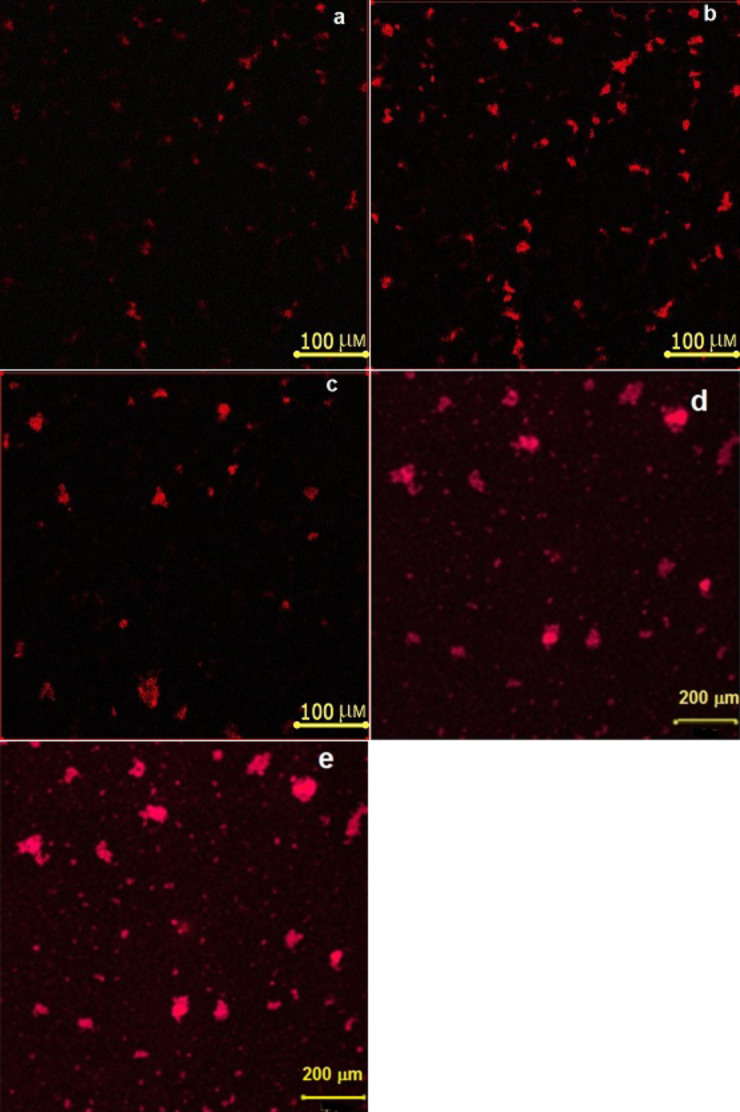
Photoluminescence images of CdSe/ZnS QDs in LC matrices obtained using a confocal microscope with a wavelength of 409 nm. Planar orientated LC cell (a) in the initial state, (b) at 0.25 V/μm (b) and (c) at 1.5 V/μm; vertical orientated LC cell (d) in the initial state and (e) with the maximal PL intensity.

Structural changes in the active LC matrix not only lead to a change in the luminescence properties of QDs, but also to the displacement of particles and a change of their size. Figure 3 shows confocal microscopy images of QDs and their aggregates in the active (a, b, c) and the passive (d, e) LC matrix. We have obtained the images in Figure 3a,d after filling the LC cells without the application of an electric field. The images in Figure 3b,e correspond to the maximal PL of QDs in the active and passive LC matrices at the electric field strength of 0.25 V/μm. The number of luminous aggregates in Figure 3c decreased. This testifies the quenching of QD luminescence in an active LC matrix at 1.5 V/μm of the electric field strength.

In the passive LC matrix, no significant changes in the particle size distribution in Figure 3d,e are observed. When the LC molecules do not reorient, QDs aggregates do not change their location. The results of the statistical analysis of the particle-size distribution in the active LC matrix are given in Table 1. The average area of QD aggregates in Figure 3a–c increases in Table 1. Its magnitude increases by about 8% at a field strength of 0.25 μV/m in the planar oriented LC cell. The elastic forces acted in the LC matrix can contribute to QD aggregation. The area of QD aggregates increased by 28% during the reorientation of LC molecules with increasing the strength of electric field applied to the LC cell.

**Table 1:** The QD size distribution in the active LC matrix.

	electrical field strength, V/μm		
	0	0.25	1.5

min area of particles, μm^2^	1.92	2.92	12.06
max area of particles, μm^2^	664.73	1197.17	1180.61
average area of particles, μm^2^	143.72	155.07	183.15

The average area of the particles in the LC cell with the vertical orientation of the molecules was about 395.92 μm^2^ (Figure 3d,e). The formation of larger agglomerates at the same weight content of QDs in the LC can lead to the steady-state quenching of luminescence in the passive LC matrix (Figure 2a).

**Figure 4:**
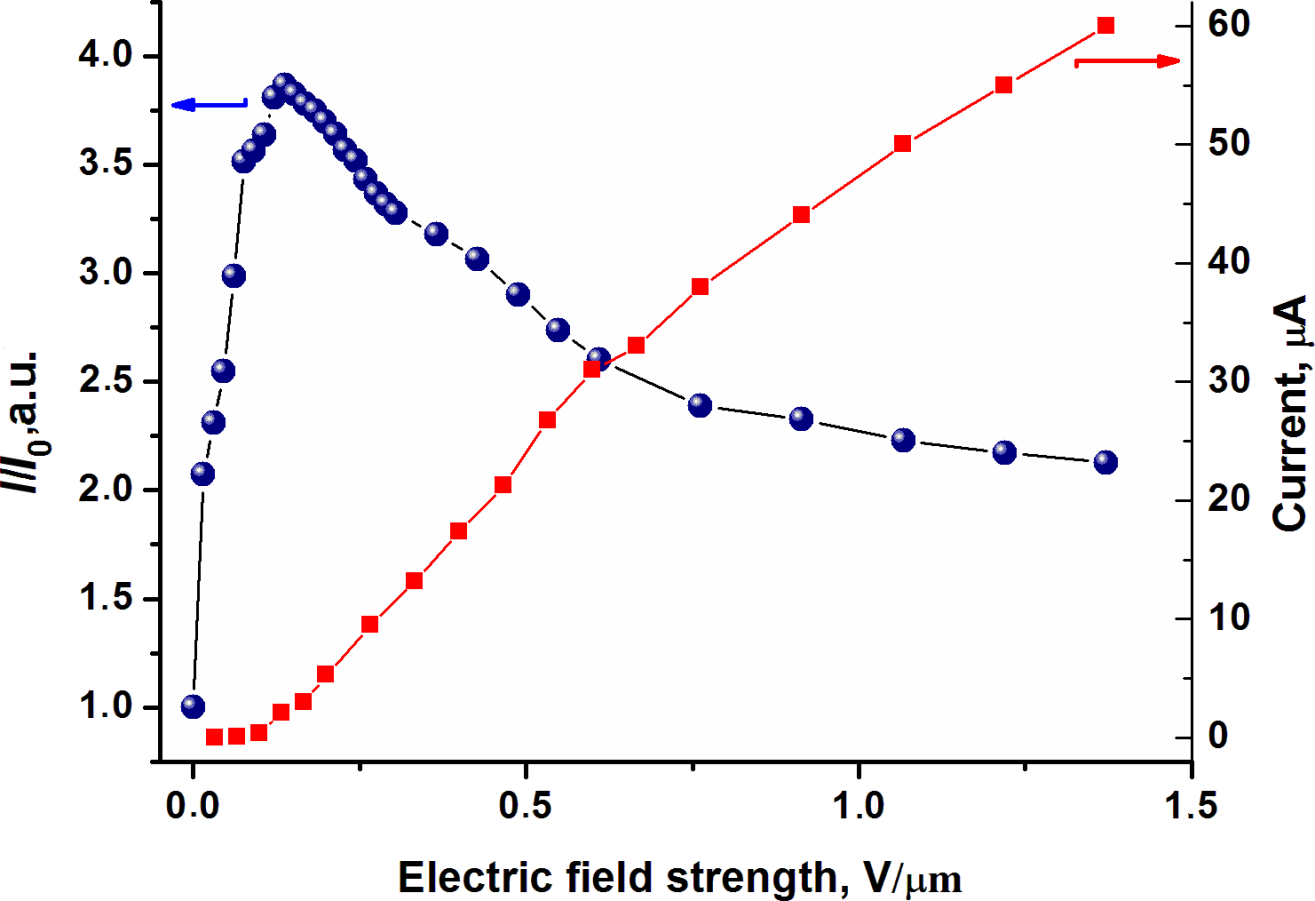
Change in the PL intensity (blue circles) and ion current (red circles) in the active LC matrix with 1 wt % CdSe/ZnS QDs as a function of the electric field strength.

The addition of CdSe/ZnS QDs in the LC leads to an increase in ionic impurities and conductivity of LCs [26–27]. The reason for this is the partial destruction of the QD shell during the mixing process and the appearance of additional slow ions in the LC. Such slow ions can be TOPO molecules with weak coordination bonds to the surface of the QDs [26]. We compared the dependencies of PL intensity and ion current on the electric field strength in the LC cell with an initially planar orientation in Figure 4.

The presence of a PL intensity peak in Figure 4 indicates two competing processes. One of them causes an increase, and a second the quenching of the luminescence. At low electric field strengths, there is no the reorientation of LC molecules, and the PL intensity of QDs increases in the absence of ion current. The quenching of QD luminescence begins with the reorientation of LC molecules in the electric field. The mobility and current of ions increase in the LC. Therefore, the influence of charge carriers on the deactivation of QDs photoexcitation is possible. In the passive LC matrix, the director aligns along the electric field vector and the position of the LC molecules and QDs do not change. When a dc electric field is applied to the LC cell, the charge carriers separate, forming double electrical layers near the interfaces. We assume that the photoexitation and quenching of the luminescence of the QDs occurs simultaneously in the passive LC matrix in contrast to the active nematic matrix. Thus, in weak electric fields up to the Fréedericksz threshold, it is possible to control the buildup of the CdSe/ZnS QDs luminescence. By changing, the strength of electric field is possible to control the quenching of QDs photoluminescence because of the reorientation of the LC molecules. However, additional studies need to explain the mechanisms of the PL intensity of CdSe/ZnS QDs buildup and its dynamic quenching in LC.

## Conclusion

We have compared changes in the luminescence of semiconductor quantum dots CdSe/ZnS with a core diameter of 5 nm and a concentration of 1 wt % in the active and passive liquid crystal matrix in electric field. The nematic with positive dielectric anisotropy and a planar orientation of LC molecules in the initial state was used as an active matrix. In a passive LC matrix, the molecules were oriented vertically and did not reorient in the electric field. We have found out that the PL intensity of QDs increases in the active LC matrix by four times and in the passive LC matrix only by 1.6-times at an electric field strength of 0.25 V/μm. Quenching of the QD luminescence occurs in the active LC matrix with a further increase in the electric field strength. In the passive LC matrix, the achieved level of PL intensity remained practically constant with increasing field strength. The decay times showed a similar dependence on the electric field strength. Size change and redistribution of QD agglomerates were observed in the active matrix. The luminescence quenching was accompanied by an increase of ion current in the active LC matrix with increasing electric field strength. To explain the mechanisms additional investigations are needed. The experimental results are of practical interest for controlling the photoluminescence QDs intensity in liquid crystals by means of an electric field.
